# Boosting effect of high-dose influenza vaccination on innate immunity among elderly

**DOI:** 10.1172/jci.insight.184128

**Published:** 2025-03-04

**Authors:** Olivia Bonduelle, Tristan Delory, Isabelle Franco-Moscardini, Marion Ghidi, Selma Bennacer, Michele Wokam, Mathieu Lenormand, Melissa Petrier, Olivier Rogeaux, Simon de Bernard, Karine Alves, Julien Nourikyan, Bruno Lina, INFLUOMICS Study group, Behazine Combadiere, Cécile Janssen

**Affiliations:** 1 Sorbonne Université, Institut National de Santé et de Recherche Médicale, Inserm U1135, Centre d’Immunologie et des Maladies Infectieuses, Cimi-Paris, Paris, France.; 2 Centre Hospitalier Annecy Genevois, Epagny Metz-Tessy, France.; 3 Centre Hospitalier Centre Hospitalier Métropole Savoie, Chambéry, France.; 4 Altrabio, Lyon, France.; 5 Centre International de Recherche en Infectiologie, Université Claude Bernard Lyon-1, INSERM U1111, CNRS, UMR5308, ENS Lyon, Université Jean Monnet de Saint-Etienne, Lyon, France.; 6 Laboratoire de Virologie, Institut des Agents Infectieux, Centre National de Référence des Virus des Infections Respiratoires, Hospices Civils de Lyon, Lyon, France.; 7 The INFLUOMICS Study group is detailed in Supplemental Acknowledgments.

**Keywords:** Immunology, Vaccines, Influenza, Innate immunity

## Abstract

**BACKGROUND:**

The high-dose quadrivalent influenza vaccine (QIV-HD) showed superior efficacy against laboratory-confirmed illness compared with the standard-dose quadrivalent influenza vaccine (QIV-SD) in randomized controlled trials with the elderly. However, specific underlying mechanism remains unclear.

**METHODS:**

This phase IV randomized controlled trial compared early innate responses induced by QIV-HD and QIV-SD in 59 individuals aged > 65 years. Systemic innate cells and gene signatures at day 0 (D0) and D1 as well as hemagglutinin inhibition antibody (HIA) titers at D0 and D21 after vaccination were assessed.

**RESULTS:**

QIV-HD elicited robust humoral response with significantly higher antibody titers and seroconversion rates than QIV-SD. At D1 after vaccination, QIV-HD recipients showed significant reduction in innate cells, including conventional DCs and NK cells, compared with QIV-SD, correlating with significantly increased HIA titers at D21. Blood transcriptomic analysis revealed greater amplitude of gene expression in the QIV-HD arm, encompassing genes related to innate immune response, IFNs, and antigen processing and presentation, and correlated with humoral responses. Interestingly, comparative analysis with a literature dataset from young adults vaccinated with influenza standard-dose vaccine highlighted strong similarities in gene expression patterns and biological pathways with the elderly vaccinated with QIV-HD.

**CONCLUSION:**

QIV-HD induces higher HIA titers than QIV-SD, a youthful boost of the innate gene expression significantly associated with high HIA titers.

**TRIAL REGISTRATION:**

EudraCT no. 2021-004573-32.

## Abstract 2

Compared to standard dose, high dose influenza vaccine in elderly revealed a greater amplitude of innate gene signature responses similar to younger adults.

## Introduction

Influenza is a contagious disease due to respiratory RNA viruses (1, 2). Vaccination with the influenza vaccines is the foremost strategy for preventing influenza and its severe complications on a global scale. Extensive evidence supports the effectiveness of influenza vaccination in diminishing morbidity and mortality associated with influenza, particularly in high-risk groups, including neonates, young children, pregnant women, and individuals aged 50 years or older (3, 4).

Despite the overall efficacy of influenza vaccination campaigns, emerging evidence indicates that vaccines may elicit diverse responses, leading to suboptimal immunity in specific populations, particularly in the elderly. Hence, a significant public health challenge lies in developing a vaccine that effectively safeguards populations with diverse and comparatively weaker immune systems than the average. Given the high mutation rate and transmissibility, it is also suggested that the next pandemic is likely to be caused by a respiratory RNA virus, including influenza (5). Understanding biological pathways leading to optimal immunization is thereby essential in this perspective. Several factors are known to influence the immune response to influenza vaccines and should be encompassed in the design of vaccine strategies: (a) host genetic factors (i.e., genetic polymorphisms); (b) host-related factors such as age, comorbidities, physiological stress, and microbiome; and (c) vaccination strategies, including mode of vaccine administration, doses, and adjuvants. Based on the findings previous studies, we proposed that the early innate immune response triggered in the hours following vaccination plays a crucial role in shaping the magnitude and effectiveness of the adaptive immune response in the weeks following vaccination (6–8). This assumption was supported by the integration of biological data obtained during influenza vaccination clinical trials. These various studies have provided valuable insights into the effect of immunization methods and vaccine formulations on the innate signature, influencing the quality of immune responses (7, 9, 10). It could also contribute to epidemic and pandemic preparedness, giving guidance for industry to develop future vaccine candidates for emerging influenza strains, including mRNA strains.

Optimal targeted strategies to improve immune responses to the vaccine in the elderly population could stem influenza-associated morbidity and mortality (4). One way is to increase the dose of HA in inactivated vaccines (11, 12), such as Efluelda (Sanofi Pasteur high-dose quadrivalent influenza vaccine [QIV-HD], 60 μg hemagglutinin [HA] per strain) for elderly population. The QIV-HD stands out as the only vaccine that has shown superior efficacy against laboratory-confirmed illness in adults aged 65 years and older. This was demonstrated in a significant pivotal randomized controlled trial, comparing it with the standard-dose QIV (QIV-SD, 15 μg HA) (12), along with established and consistent performances over many consecutive influenza seasons (13).

In addition to the direct clinical benefits of QIV-HD vaccine, the immunological responses of high-dose versus standard-dose vaccines were documented in a recent systematic review (14). High-dose influenza vaccines have demonstrated the ability to elicit elevated hemagglutination inhibition antibody (HIA) titers, increased neutralization, and higher antineuraminidase titers. However, their effects on other facets of immunity remain unknown. Further studies delving into the contributions of various immune cells to this improved immune response could offer valuable insights, particularly regarding the vaccine’s application in special groups at risk of infection. To enhance vaccination strategies, particularly for elderly individuals, a deeper understanding of the intricate molecular mechanisms and interconnected networks regulating innate and adaptive immunity induced by high-dose vaccines is essential (15).

We advocate for holistic systems vaccinology approaches that concurrently consider both innate and adaptive immunity. This comprehensive perspective enables us to gain a global understanding of the response to an influenza vaccine. The foundation of systems vaccinology rests on 2 core objectives: first, unveiling new mechanisms involved in vaccination, and second, identifying novel biomarkers that can distinguish between responders and nonresponders to vaccines. Ultimately, these approaches will facilitate the identification of early molecular and cellular factors that may contribute to the enhanced clinical protection offered by high-dose vaccines in the days following vaccination. This knowledge will enable tailoring vaccination strategies to specific populations, ensuring the right dose, for the right population, and employing the right delivery strategy.

One of our approaches is based on measurement of variations in innate immune responses in the blood during the 24 hours following vaccination (7, 9, 10). We can leverage this data to assess the intensity of immune responses linked to key innate immune gene signatures and compare them to gene signatures documented in existing literature (7, 9, 10).

We hypothesize that the dose of QIV HA can affect the intensity and quality of innate immunity and, subsequently, the humoral responses. In this phase IV open-label randomized (1:1 ratio) versus active-controlled multicenter trial, we aim at comparing the effect of QIV-HD versus QIV-SD in patients aged 65 years or older, on the 2 components of immune response: early innate and adaptive immunity. The relative evolution of (a) the early blood molecular (transcriptome) and the cellular (blood phenotyping) signatures 1 day (D1) after vaccination, and (b) the late adaptive immunity D21 after vaccination with either QIV-HD or QIV-SD vaccinees during the 2021/2022 influenza season, were compared with baseline values.

## Results

### Study population and HIA titers.

**Figure 1:**
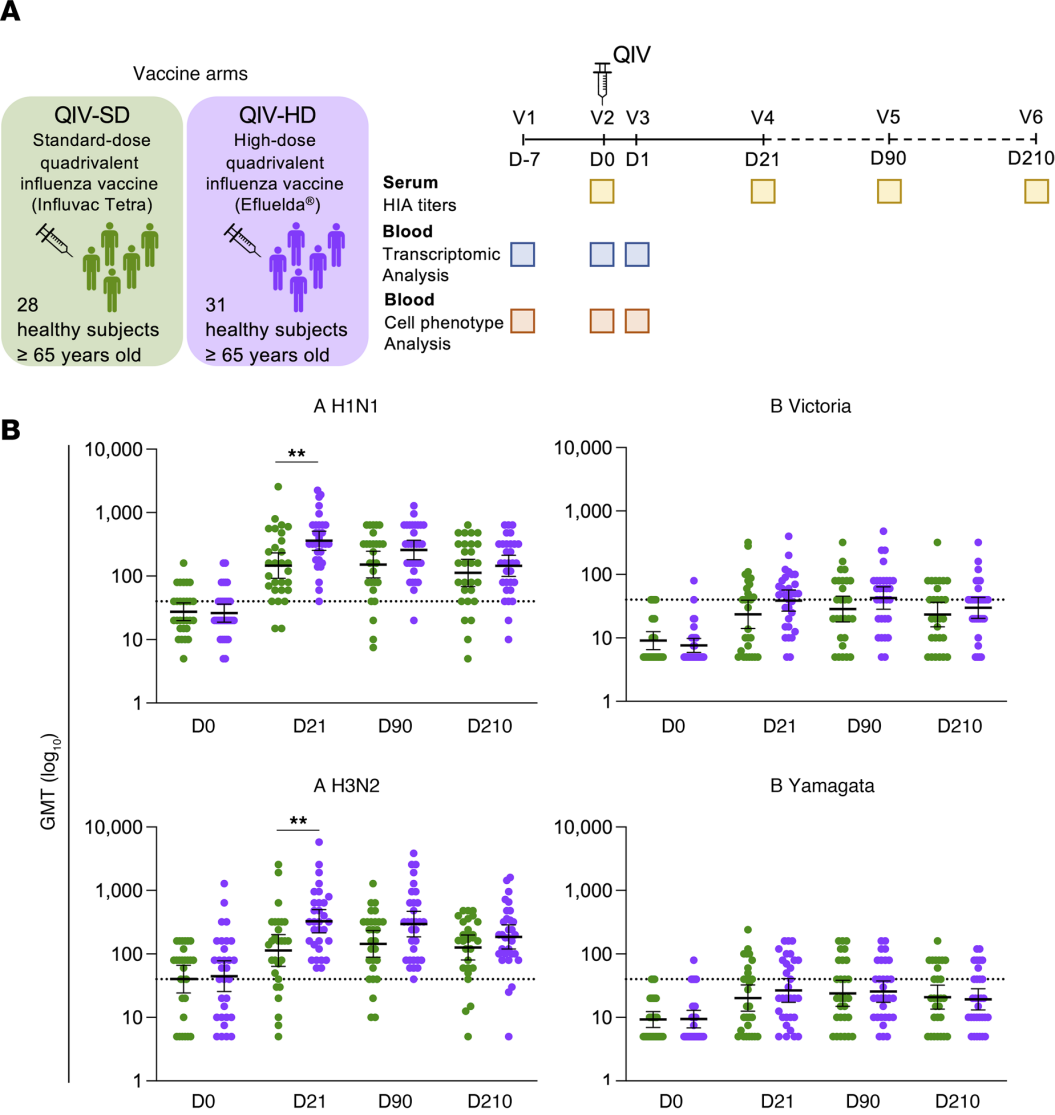
Study protocol and immunogenicity of QIV-HD compared with QIV-SD in adults ≥ 65 years old. (**A**) Schematic overview of experiment with kinetics of biological sample collection including serum collection and whole blood cells for transcriptomic and innate cell phenotype analyses. (**B**) Dot plots present geometric mean titers (GMT) with 95% CI of anti–A H1N1 hemagglutination inhibition antibody (HIA) titers, anti–A H3N2 HIA titers, anti–B Victoria HIA titers, and anti–B Yamagata HIA titers at D0, D21, D90, and D210 after QIV-SD (green) or QIV-HD (violet) vaccination. The horizontal line represents the seroprotection cut off with HIA titers at 40. Wilcoxon tests and Fisher’s exact tests were performed to compare the characteristics of the 2 groups (**P <0.01).

A total of 59 participants with a signed informed consent were included, randomized. Of them, 31 were assigned to QIV-HD arm and 28 to QIV-SD arm. The flowchart diagram is presented in Figure 1A and Supplemental Figure 1 (supplemental material available online with this article; https://doi.org/10.1172/jci.insight.184128DS1). Biological and clinical data were complete for all patients (Supplemental Figure 1). Participant characteristics were similar between both arms. The median age of the participants was 70 years, and females were 42% (*n* = 25) of the study participants (Supplemental Table 1). During the 2020/2021 winter season, 83% were vaccinated against influenza using 1 dose of QIV-SD, and 100% patients received 2 doses or more of SARS-CoV-2 vaccine in a median of 2 months prior trial participation (Supplemental Table 2). Safety between arms was similar, with 5 serious adverse events occurred in 4 patients. One of them was a suspected unexpected serious adverse reaction 120 days after QIV-HD vaccination, and 4 were considered not to be related to the vaccine or the study procedure by the investigators and the sponsor (Supplemental Table 3).

We first compared the immunogenicity of QIV-HD versus QIV-SD by measurement of HIA titers in the study population. Results are displayed in Figure 1B and Supplemental Table 4. QIV-HD induced significantly higher geometric mean titers (GMTs), seroprotection, and seroconversion rates against influenza A strains (A/H1N1 and A/H3N2) D21 after vaccination (*P* < 0.01). However, HIA titers were not sustained until D210 after vaccination. The immune responses against influenza strain B were less effective in both study arms, yet higher seroconversion rate was observed against influenza B/Victoria strain when receiving QIV-HD compared with QIV-SD.

Our study validated elevated immunogenicity of QIV-HD compared with QIV-SD in elderly patients aged > 65 years.

### Early whole blood gene expression following QIV-HD compared with QIV-SD vaccination in the elderly population.

Our hypothesis posits that the robust humoral responses observed after QIV-HD administration could be attributed to modulation of innate responses at gene expression levels. Initially, we employed transcriptomic methods to assess the early innate immunity’s quality, irrespective of the outcomes in adaptive immunity. Through this, we identified a distinct “gene expression signature” after vaccination by comparing gene expression within the initial 24 hours following vaccination (from D1 to D0) across different trial arms.

**Figure 2:**
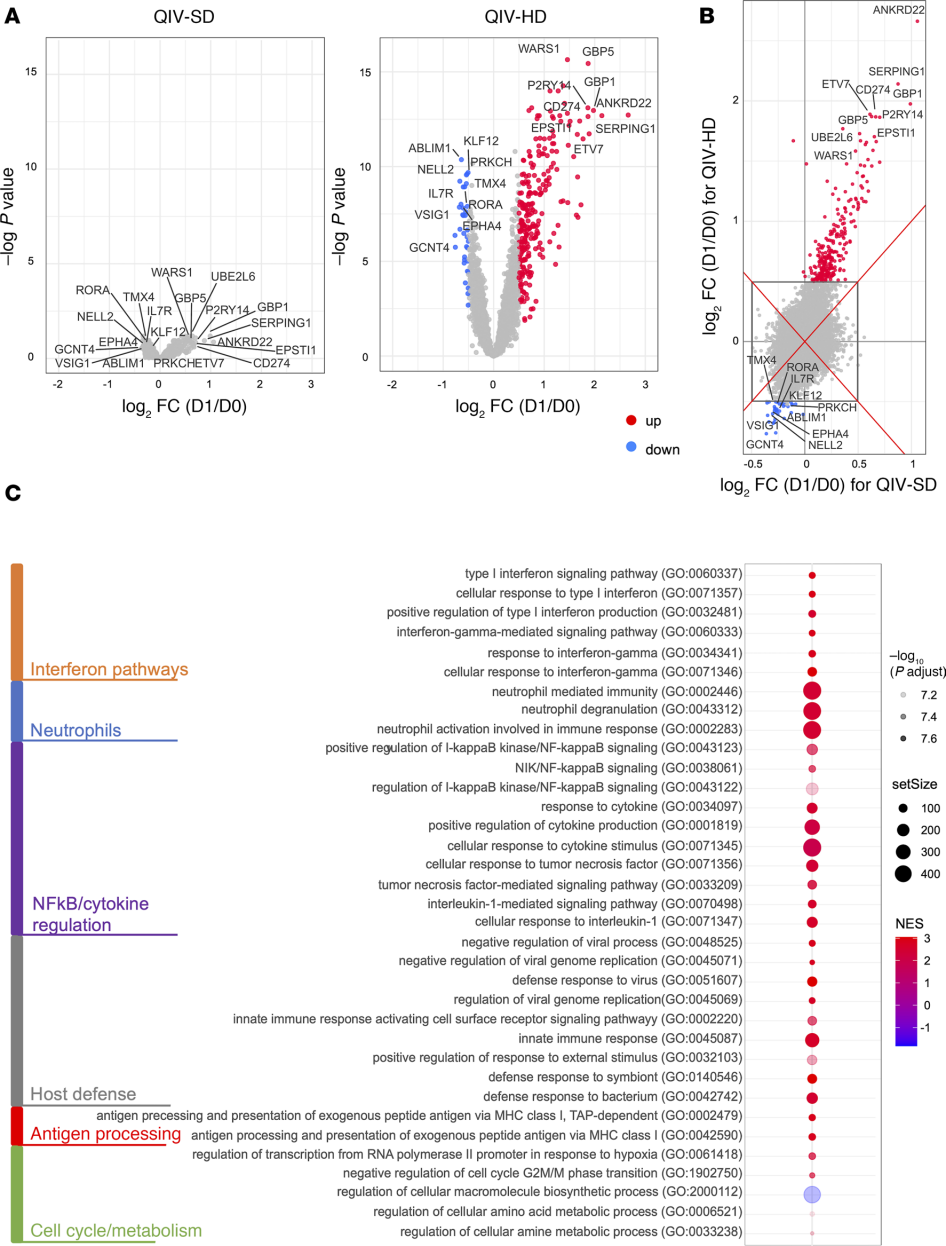
Differential expression genes by transcriptomic analysis at D1 after vaccination in QIV-HD and QIV-SD arms. (**A**) Left panel: Volcano plot of differentially expressed genes (DEG) (D1/D0) of arm QIV-SD. Significance is defined by a FDR adjusted *P* < 0.05 and a FC > 1.41 (red; upregulated) or < –1.41 (blue; downregulated). Right panel: Volcano plot of DEG: (D1/D0) of arm QIV-HD. The vertical black lines delimit the 1.41-FC effects. (**B**) Differential FC (D1/D0) comparison of gene expression between QIV-SD and QIV-HD groups. The black square delimits the 1.41-FC effects. Significance is defined by an FDR adjusted *P* <0.05 and a FC > 1.41 (red; upregulated) or < –1.41 (blue; downregulated). (**C**) Biological processes enriched by the DEG in the QIV-HD. DEG were ranked by their value of FC (*P* <0.05), and the GSEA algorithm using Gene Ontology – Biological Processes was performed to extract functional information from gene expression data.

Gene expression 7 days before (D–7) vaccination, and upon immunization (D0) was similar between treatment arms (data not shown). In the 24 hours following vaccination (D1), compared with D0, we observed strong modifications in the blood transcriptome with each vaccine, and we identified genes that were differentially expressed (D1/D0) with each vaccine, shown in Figure 2, A and B. The statistical analyses showed that modification in gene expression was induced by both vaccines, yet to a much greater and significant extent in patients vaccinated with QIV-HD. We did not detect significant differentially expressed genes (DEG) (adjusted *P* < 0.05 and with absolute fold-change [FC] of 1.41), at D1 in the QIV-SD arm, while we found 293 DEG in the QIV-HD arm, suggesting a higher intensity of innate signature in elderly patients vaccinated with high-dose influenza vaccine. The list of gens is reported in Supplemental Table 5. Genes were similarly up- or downregulated by vaccines, with higher intensity among patients receiving QIV-HD compared with QIV-SD (Figure 2B).

Although to different extents in terms of FC and *P* values, QIV-HD vaccines induced expression of genes involved in the same biological pathways, as observed in the enrichment analysis performed by Gene Set Enrichment Analysis (GSEA) algorithm using the Gene Ontology (GO) database (Figure 2C). Among the biological activated pathways, we observe those related to “innate immune response”, “responses to virus and to interferons (IFNs)”, and “antigen processing and presentation”. Indeed, among the top DEG (lowest *P* values and highest FC), many were known to be involved in these major biological pathways, such as UBE2L6 (Class I MHC antigen processing and presentation), GBP5 and GBP1 (IFN inducible GTPases), SERPING1 (regulation of complement cascade), and ANKRD22 (antiviral activity) (Supplemental Table 6).

### Gene signature of H3N2 HIA titers following QIV-HD vaccination in elderly individuals.

**Figure 3:**
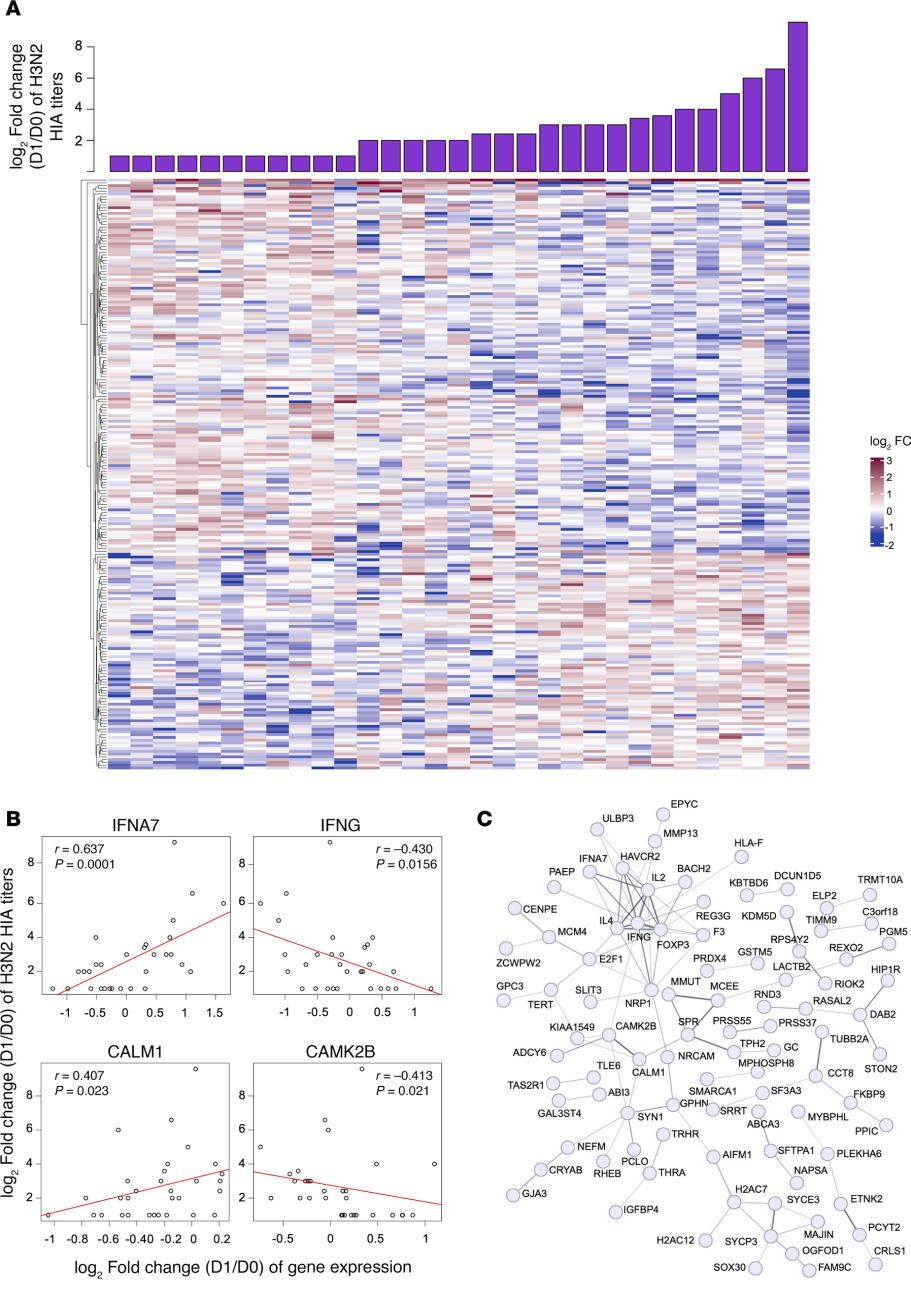
Early innate gene signature following QIV-HD vaccination in elderly. (**A**) Heatmap of the log_2_ FC values of the 213 genes correlated with the log_2_ FC of anti–A H3N2 HIA titers. The graph displays the values for each patient, ordered by increasing values in the log_2_ of antibody titers, which are displayed in the bar plot (Spearman correlation, *P* < 0.05 and *r* > 0.4). (**B**) Correlation plots of the log_2_ FC of H3N2 HIA titers (*y* axis) and the log_2_ FC of some of the top genes correlated with them (*x* axis). (**C**) STRING pathway analysis of genes correlated with the log_2_ FC of anti–A H3N2 HIA titers, without disconnected nodes in the network. Edges represent protein-protein associations with minimum required interaction score of 0.4 edge confidence (line thickness indicates the strength of data support).

We sought to define an early innate gene signature associated with HIA titers following QIV-HD influenza immunization. Considering the intensity of humoral responses to A/H3N2 HIA titers in our dataset, and consistent with previous studies on the dominance of A/H3N2 HIA titers (16), we observed that the log_2_ FC of 213 genes correlated with the log_2_ FC of A/H3N2 HIA titers (Supplemental Table 7). We applied hierarchical clustering to the expression profile of these 213 genes for which the log_2_ FC was found significantly correlated (*P* < 0.05 and *r* > 0.4) with the log_2_ FC of A/H3N2 HIA titers. As shown in Figure 3A, we observed their expression among volunteers with increasing values of antibody response against H3N2 virus strain (Figure 3A and Supplemental Table 7). We found genes that are involved with antiviral and IFN signaling responses as well as other innate immunity pathways. In Figure 3B, we represented a few examples of genes (IFNA7, IFNG, CALM1, and CAMK2B) with the expression levels that are positively or negatively correlated with log_2_ FC of A/H3N2 HIA titers. STRING analysis (https://string-db.org/) showed their biological link (Figure 3C). CALM1 and CAMK2 belongs to the calmodulin pathway, as well as CAMK4, which was described by Nakaya et al. (17) and negatively correlated with Ab responses at D3 after vaccination.

Our study thereby validates previous findings on an early innate gene signature linked to HIA titers following QIV-HD with pronounced effect in elderly individuals.

### Early whole blood cellular variation following QIV-HD compared with QIV-SD vaccination in the elderly population.

**Figure 4:**
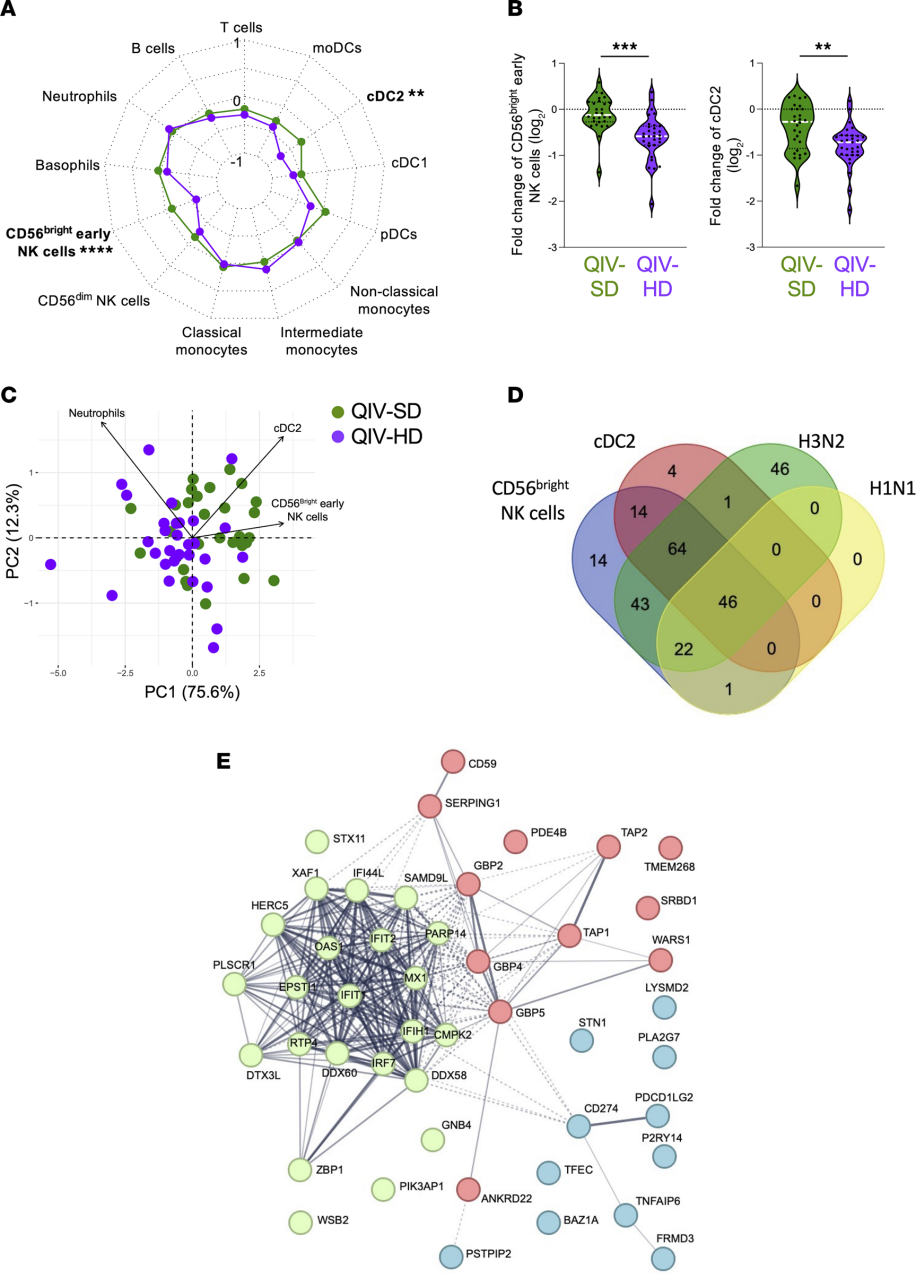
Innate cellular responses following QIV-HD and QIV-SD vaccination in elderly. (**A**) Radar chart presents the 1 and –1 FC (log_2_) for participant groups. The medians of each blood cell population (T and B cells; neutrophils; basophils; CD56^bright^ early and CD56^dim^ NK cells; classical, intermediate, and nonclassical monocytes; pDCs, cDC1, cDC2, and moDCs) are presented for QIV-SD (green) and QIV-HD (violet) groups. (**B**) Violin plot of log_2_ FC (D1/D0) are represented for CD56^bright^ early NK cells and cDC2 for 2 vaccinated groups. (**C**) Principal component analysis (PCA) representation of QIV-SD (green) and QIV-HD (violet) groups based on induced blood cell populations at D1/D0. Arrows indicate the prominent parameter distinguishing features (neutrophils, CD56^bright^ early NK cells and cDC2). (**D**) Venn diagram of common DEG correlated with anti–A H3N2 (green) and –A H1N1 (yellow) HIA titers, CD56^bright^ early NK cell FC (blue), and cDC2 FC (red). (**E**) STRING pathway analysis (with 3 K-means clustering represented by 3 node colors) of 46 genes are in common between all these 4 parameters. Statistical analyses were performed with 1-way ANOVA and Bonferroni’s multiple-comparison tests, and indicated as adjusted *P* values. **P < 0.01, ****P < 0.0001.

We analyzed the abundance of different blood cell subpopulation at D1 after vaccination as indicated in the gating strategy (Supplemental Figure 2). The results are represented in percentage of CD45^+^ cells, excluding eosinophils. Different blood cell populations were identified using conventional surface markers: T and B cells; neutrophils; basophils; CD56^bright^ early and CD56^dim^ NK cells; classical, intermediate, and nonclassical monocytes; and plasmacytoid-, type 1 and 2 conventional-, and monocyte-derived DCs (pDC, cDC1, cDC2, and moDC, respectively). These blood cell populations remained were stable before (D–7) and upon randomization (D0) (Supplemental Figure 3). To compare the modulation of blood cell populations following vaccination (D1) by trial arm, we normalized and represented the FC differences (D1/D0) by arm using a radar chart (Figure 4A). Two main populations were significantly lowered in the QIV-HD arm 24 hours after vaccination, compared with the QIV-SD arm (Figure 4, A and B): cDC2 and early CD56^bright^ NK cells (adjusted *P* = 0.03 and < 0.0001, respectively), whereas other circulating blood cell populations remained stable (Supplemental Figure 4). The percentages of cDC2 and CD56^bright^ NK cells are more strongly decreased for the QIV-HD group compared with the QIV-SD group (Figure 4B). Only neutrophils, major blood cells, were increase in QIV-HD group but not significantly (FC = 0.2363; Figure 4A). Principal component analysis (PCA) displayed in Figure 4C visualized FC of neutrophils, cDC2, and early CD56^bright^ NK cells by trial arm. This reduction considers approximatively for 87.9% of the variation of the initial information, segregating QIV-HD and QIV-SD vaccinated individuals according to neutrophils, cDC2 and CD56^bright^ NK cells.

We then investigated gene signature correlated with differential blood circulation of cDC2 and CD56^bright^ NK cells. Among the 293 DEG identified for QIV-HD (Figure 2B and Supplemental Table 5), we found 129 DEG correlated with cDC2 FC and 204 DEG with CD56^bright^ NK cell FC (Supplemental Tables 8 and 9, respectively). The Venn diagram displayed in Figure 4D depict differentially up- and downregulated genes at D1 correlated with cDC2, CD56^bright^ NK cell population, and influenza H1N1 and H3N2 HIA titers. Common gene are represented at the interactions in the Venn diagram (Figure 4D). We found 46 common genes between these 4 variables, including genes that are associated with IFN signaling pathways as represented in a functional protein association network (https://string-db.org/) (Figure 4E). These results suggest that perturbation of cells and gene expression in the 24 hours following vaccination (D1) are a trademark of differences between HD and SD influenza vaccination.

### Similarities of gene signatures associated with early innate response modules among elderly individuals vaccinated with QIV-HD and young adults vaccinated with trivalent inactivated vaccine-standard dose.

**Figure 5:**
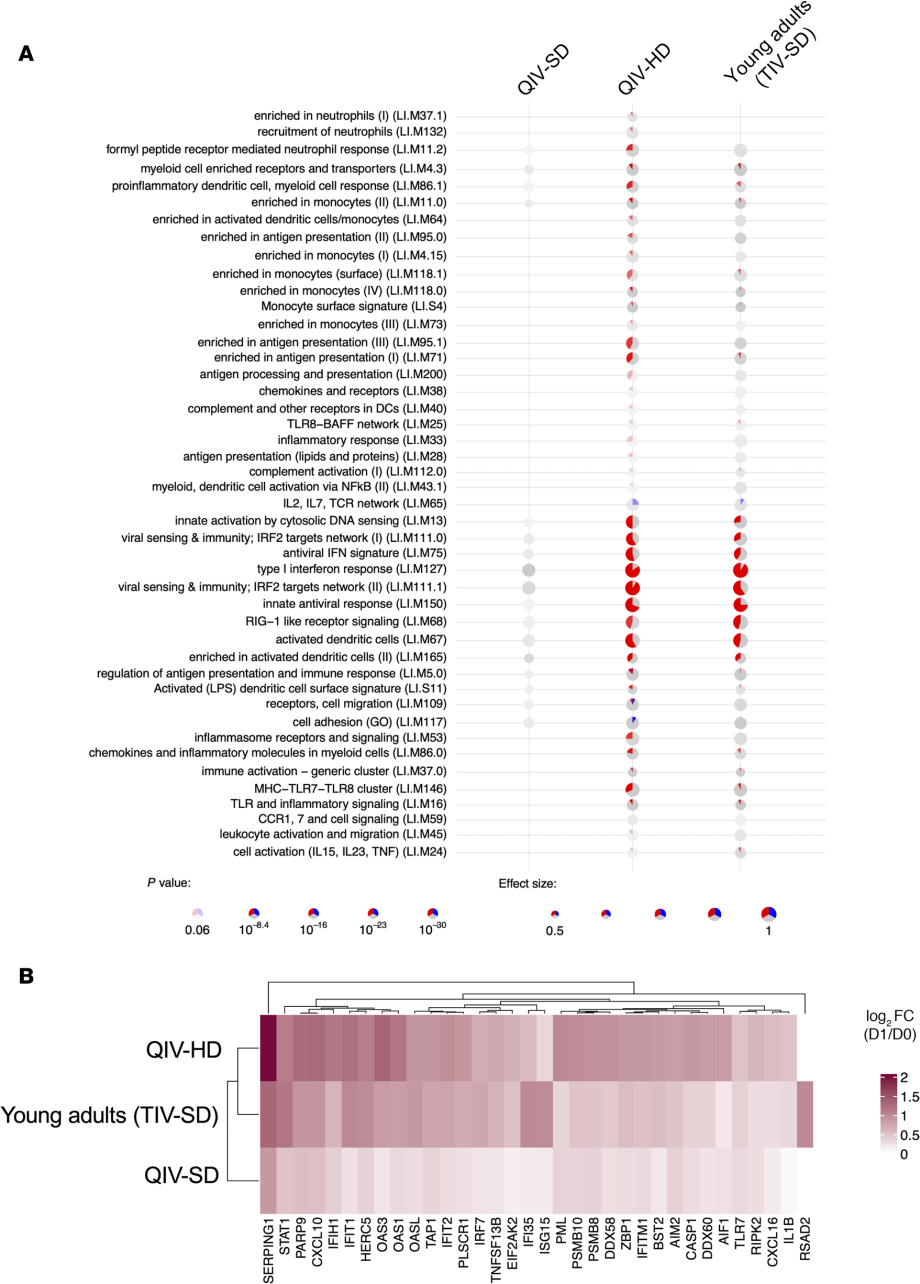
Significant up- and downregulated gene associated with early innate response modules in elderly individuals vaccinated with QIV-SD and QIV-HD compared with young adults vaccinated with TIV-SD. (**A**) The *tmod* enrichment analysis for QIV-SD, QIV-HD and young adults vaccinated with TIV-SD. From the list of genes provided by the *limma* R package, genes were ranked by their *P* values, and the enriched blood transcription modules were obtained by the CERNO test. The effect size is proportional to the size of the pie, while the adjusted *P* value is proportional to color intensity. Within each pie, the proportion of significantly upregulated and downregulated genes is shown in red and blue, respectively. The gray portion of the pie represents genes that are not significantly differentially regulated. (**B**) Heatmap of the log_2_ FC of the biomarkers of immune response that belong to the IFN signaling pathway, comparing the QIV-SD and QIV-HD arms with the meta-analysis of studies with young adults vaccinated with TIV-SD. The combining approach from the MetavolcanoR package was used to summarize the FC of the studies based on the mean of the values, while the *P* value was combined using the Fisher method.

The gene signature (293 DEG) that we identified among elderly individuals vaccinated with QIV-HD was involved in the antiviral and IFN signaling, monocyte/inflammation, antigen-processing and presentation (Figure 5A and Supplemental Table 10), and NK cell activation (Supplemental Figure 5 and Supplemental Table 11). Interestingly, this signature of humoral responses reflects the overall pattern of immune activation observed following vaccination in young adults as published previously (9, 18–20). We explored the extent of the overlap between gene signature observed in elderly receiving QIV-HD vaccine to that of the established signature in young adults, using the MetavolcanoR package. This allowed us to integrate data from various gene expression studies accessible in public repositories, all generated using similar transcriptomic arrays. Specifically, we focused on 3 studies that compared gene expression on the first day after influenza vaccination against D0 in young adults (18–20): GSE30101, GSE48023, and GSE48018 data sets. We observed that QIV-HD led to the enrichment of biological pathways described above with a significant proportion of upregulated and downregulated genes likely to the gene ensemble of young adult cohorts vaccinated with trivalent standard dose influenza vaccine (TIV-SD). We did not observe this comparability of signal intensity with QIV-SD vaccination (Figure 5A). Since we observed the enrichment of many IFN-related pathways, and the correlation of some of the genes from these modules with the antibody response in the elderly cohort (Figure 3), we next sought to inspect the differences in the expression of these genes among groups. The heatmap in the figure (Figure 5B) represents the average of the FC values for each of these genes for each cohort (QIV-HD, QIV-SD, and TIV-SD [young cohort]). We observe that, indeed, the perturbations in the expression of these genes in the QIV-HD (elderly) and the TIV-SD (young adults) are similar, while the QIV-SD (elderly) presented minor upregulation of these gens. In fact, some of the genes, such as IFI35, RSAD2, and ISG15, presented the highest FC average in the elderly group that received the QIV-HD.

In summary, the administration of HD influenza vaccine in elderly induce elevated innate gene expression in correlation with HIA titers, comparable with that observed in younger adults administered with TIV-SD.

## Discussion

In this study, we conducted a comprehensive analysis of the immune responses and safety of the QIV-HD compared with the QIV-SD in individuals aged > 65 years. Our findings contribute to the growing body of knowledge on vaccine-induced innate and adaptive immune responses. Importantly, our randomized study includes a variety of data, without missingness, ensuring the analysis integrity and the draft of causality. The present study possesses several noteworthy strengths that bolster its scientific rigor and contribute to the validity of its findings. This investigation was conducted as a randomized active controlled trial, a robust study design allowing to balance all factors known or unknown to influence the immune response upon influenza vaccination, including host genetic factors and hostrelated factors, and thereby to draft causal inference between vaccine allocated to elderly and the evolution of biological parameters. The study implemented stringent exclusion criteria, an instrumental aspect in bolstering its internal validity. These criteria allowed to exclude individuals with recent vaccine exposure or influenza-like illness (ILI), including infections due to RNA respiratory viruses. This meticulous exclusion process alongside randomization, serves to eliminate potential sources of bias and ensures that observed changes in biological parameters are attributable to the administered vaccine rather than external factors. An additional strength of this study is the absence of attrition which enhances the reliability of the results and supports the robustness of the conclusions drawn. Indeed, attrition or loss of participants over the course of the study can introduce bias and compromise the statistical power of a study.

First and foremost, our study reaffirms the safety of both QIV-HD and QIV-SD in individuals older than 65. This aligns with the broader body of research that has consistently demonstrated the safety of these vaccines. Our results provide further assurance to healthcare providers and individuals considering influenza vaccination in this age group. It also confirms that QIV-HD vaccines induce a significantly higher elevation of HIA titers than QIV-SD, 21 days after vaccination. This observation suggests that QIV-HD induces a more robust adaptive immune response compared with the standard dose, which is highly relevant in the context of influenza protection in the elderly population. However, we assessed a low number of individuals and did not observe a maintenance of humoral responses over time (D210) as previously proposed (21). The high-dose influenza vaccine is the only influenza vaccine having demonstrated superior efficacy against ILI relative to the standard-dose vaccine through a large randomized controlled trial in adults 65 years of age and older (3, 12, 22), along with established and consistent performance over many consecutive seasons (13).

Our study explored changes in the frequencies of specific NK and DC subpopulations following vaccination. Notably, the CD56^bright^ NK cells and cDC2 showed significant depletion in the blood, suggesting migration to tissues such as the vaccination site and lymphoid tissues. CD56^bright^ NK cells, less terminally differentiated than their CD56^dim^ counterparts, are efficient cytokine producers and can become cytotoxic upon activation, contributing to both innate and adaptive immune responses (23, 24).

They produce more IFN-γ, potentially influencing IFN signaling pathways (24). We found 46 common genes between all variables (cDC2, CD56^bright^ NK, A/H1N1 and A/H3N2 HIA titers) including genes that are associated with IFN signaling pathways as represented in a functional protein association network. A study by Marquardt et al. using the yellow fever virus (YFV) 17D vaccine as a model found that YFV induced a robust NK cell response, with early activation and peak function at D6, followed by a delayed peak in proliferation at D10 (25). This research highlights the role of NK cells in viral infections and their implications for vaccine development. This research contributes to our understanding of the role of NK cells in viral infections and could have implications for the development of more effective vaccines.

In addition, several phenotypic analyses of NK cells showed that L-selectin is uniquely expressed on a subset of resting human NK cells (CD56^bright^). Notably, CD56^bright^ NK cells expressed L-selectin at a higher density than all other peripheral blood leukocytes. It was found that NK activation by PMA, IL-2, IL-15, or TGF-β downregulated L-selectin on the CD56^bright^ NK subset, while increased L-selectin levels were observed in both the CD56^bright^ and CD56^dim^ NK subsets in response to IL-12, IL-10, or IFN-α (26). This finding would favor the migratory extravasation capacity of these cells into tissues.

The combination of MPL and QS-21 in AS01 induced strong recruitment of various DC subsets, including CD26^+^XCR1^+^ cDC1, CD26^+^CD172^+^ cDC2, and a CCR2-dependent CD64-expressing inflammatory cDC2 subset, compared with antigen alone. These findings align with our study’s results, showing the involvement of cDC2 in processing exogenous antigens for MHC class II presentation, crucial for immune response induction (27).

Despite these insights, the role of these cells in influenza vaccinations for elderly individuals remains unclear. Stimulating these cells in older individuals could advance vaccine development and potentially rejuvenate aging immune systems, addressing the increased susceptibility of the elderly to infectious diseases like influenza. This area of research is vital for improving vaccine efficacy in older populations.

Our study validated earlier findings on innate gene signatures linked to HIA titers following QIV-SD but observed a more pronounced effect following QIV-HD influenza immunization. Among early gene expression, we found that IFNA7, IFNG, CALM1, and CAMK2 had expression levels that are positively or negatively correlated with log_2_ FC of A/H3N2 HIA titers. CALM1 and CAMK2 belongs to the calmodulin pathway, as well as CAMK4, which was described by Nakaya et al. (17) and negatively correlated with Ab responses at D3 after vaccination. Our study aligns with several studies that have delved into the genomic aspects of vaccine response. Bucasas et al. (18) highlighted the rapid upregulation of genes involved in IFN signaling and antigen processing upon immunization in young individuals. Franco et al. (19) shed light on the genetic determinants of the human response to influenza vaccination, particularly genes involved in membrane trafficking and antigen processing. Obermoser et al. (20) pointed to the dominance of IFN and inflammation signatures following different vaccine types, including influenza. These studies provide valuable context for our findings, underscoring the complex interplay of genetics and innate immune responses to vaccination. Kazmin et al. (28) explored the transcriptomic signature of type 1 IFN responses and the role of adjuvants in vaccine-induced innate immunity. Hagan et al. (29) analyzed transcriptional data across various vaccines and identified signatures of innate immunity induced by vaccination. Indeed, our study shows that primary vaccination induced a persistent transcriptional signature of innate immunity, and booster vaccination induced a transcriptional signature of an enhanced memory-like innate response. Considering that influenza vaccination using QIV or TIV are booster vaccinations, high-dose vaccines might enhance innate memory-like signatures including IFN signaling and antigen-presentation pathways, which may relate to the observed differences in immune responses between QIV-HD and QIV-SD. This aligns with our observation of enhanced innate immune signatures in QIV-HD recipients. Nakaya et al. (9) identified gene signatures associated with enhanced innate and adaptive responses to influenza vaccination. The concept of innate memory and the potential for vaccines to induce nonspecific protection is an emerging area of interest, as highlighted by Taks et al. (30). Our findings may contribute to the ongoing discussion of whether vaccine-induced innate immune responses can be harnessed for broader immunological benefits (24). While Hagan et al. (29) did not specifically investigate influenza vaccines, the concept of asynchronous responses across vaccines is relevant to our study. Future research could explore the precise nature of the asynchrony in immune responses between QIV-HD and QIV-SD recipients.

Interestingly, our work defines a potentially novel aspect of high-dose influenza vaccination in the elderly population by demonstrating similarities in innate signature observed in young adults receiving trivalent standard dose vaccine. Using these powerful tools to bring innovation for more potent vaccines in the elderly population is striking. Indeed, determining mechanisms of immune responses regarding the dose of vaccine in a particular “fragile” population will not only help in improvement of influenza vaccination uptake but also to revisit the vaccination strategies used in elderly population (31).

The Pulendran group (32) demonstrated that vaccination leads to persistent epigenomic remodeling of the innate immune system, pointing to the potential of adjuvants like AS03 to influence epigenetic responses. Finally, we can observe these results in the light of epigenetic analysis, as demonstrated recently (33). Indeed, short-term epigenetic memory in innate immune cells following mRNA-mediated vaccination is a phenomenon that could have implications for understanding the duration and persistence of vaccine-induced immune responses. Further investigations are needed to explore this question, particularly when increasing the vaccine dose. Comparing the innate and adaptive immunity in the elderly between high-dose and new mRNA vaccines against influenza would also be intriguing. Of note, the high-dose flu vaccine, which is derived from the influenza virus, contains single-stranded RNA with 4 times more HA than the standard dose. As a result, high-dose formulations are likely to contain more influenza viral RNA than standard-dose formulations. Despite our efforts, we were unable to find any literature detailing the manufacturing process of the high-dose influenza vaccine. It’s also worth noting that the viral RNA constraints of the high dose may contribute to its adjuvant effect, potentially enhancing immune system stimulation.

### Limitation of our study.

This study, while offering valuable insights into the immune response to influenza vaccination, also exhibits certain limitations that warrant consideration. The number of participants included in this study is relatively small. A larger sample size would enhance the statistical power and precision of the results. Although the study is randomized and endpoints are objective and less susceptible to measurement, the absence of blinding with double dummy remains a limitation. We limited our kinetic study to D1 and did not collect cells at different time points to assess innate and adaptive parameters. Our study primarily focuses on the kinetics of the humoral immune response and lacks particularly investigations on T cell responses. While these aspects are crucial, a more comprehensive examination of cellular immunity B and T cell subsets could provide a more nuanced understanding of the vaccine’s effects. Indeed, high-dose vaccine significantly increased plasmablast (antibody-secreting cells [ASCs]) responses at D7 after vaccination, which might be related to elevated innate responses (34).

The study lacks data points for the kinetic assessment of innate immune responses, possibly limiting our ability to capture critical modulation of the innate immune response over time. This affects the comprehensive characterization of vaccine-induced immunity, especially in the differentiation between high-dose and standard-dose vaccines.

In conclusion, our study highlights significant differences in intensity of immune responses, particularly in the induction of HIA titers and innate immune signatures. These findings have broader implications for the development of vaccines suitable for older populations and warrant further investigation in the long term and into the mechanisms underlying these differential responses.

## Methods

### Sex as a biological variable.

Both male and female (39% for QIV-SD group and 45% for QIV-HD group) participants were enrolled in this research study. Sex variable was not considered as a biological variable.

### Clinical protocol.

This study is a phase IV, randomized (1:1 ratio, blocks of various size, electronic allocation), open-label, active-controlled, multicenter trial enrolling adults aged > 65 years to evaluate the different mechanisms of the immunological action of QIV-HD (Efluelda) compared with QIV-SD (InfluvacTetra). Individuals enrolled did not (a) receive vaccination against influenza for 2021/22 season; (b) receive other vaccine injection in the 4 weeks preceding randomization (including SARS-CoV-2); (c) plan to receive any vaccine (including SARS-CoV-2) in the 24 hours following randomization; (d) have ongoing immunosuppressive therapy or other active immunodeficiency; or (e) have ILI symptoms, including because of COVID-19, in the 4 weeks before randomization.

Among the patients enrolled in the study, 60 were eligible and 1 was excluded (patient 02-070-CM) before randomization due to being diagnosed with an active high-grade lymphoma (noninclusion criteria). QIV-HD/Efluelda is an inactivated influenza vaccine containing 60 μg of HA (i.e., 4 times the antigen quantities contained in standard dose influenza vaccine) of each of the A strains (A/H1N1 and A/H3N2) and B strains (Victoria lineage and Yamagata lineage). QIV-SD/InfluvacTetra is an inactivated influenza vaccine containing 15 μg of HA for the same A and B strains as Efluelda. These vaccines were authorized in Europe for 2021/22 influenza season (https://www.medicines.org.uk/emc/product/100678/smpc and https://www.ema.europa.eu/en/medicines/human/paediatric-investigation-plans/emea-001782-pip01-15-m03). The formulation of these vaccines follows the seasonal influenza World Health Organization (WHO) and European Union recommendations.

Upon randomization (D0), patients were allocated to receive either immediate QIV-HD (*n* = 31) or immediate QIV-SD (*n* = 28). Patients underwent 6 trial visits from 7 days before randomization to 210 days after vaccination (Supplemental Figure 1). Blood samples were drawn at each visit. A diary was given to study participants, allowing them to report any unexpected event, including local reactions, systemic signs or symptoms, ILIs, or the intake of any unusual treatment.

### HIA assay.

We measured serum antibodies (frozen sample of serum from blood collected on dry tubes) against the 4 influenza viral strains contained in the 2021/2022 influenza vaccine at each trial visit using microtiter HIA assays. Briefly, after treatment with a receptor-destroying enzyme, serial 2-fold dilutions of serum (starting at 1:10) were tested against 4 HA units of antigen in human O^+^Rh^−^ RBCs. The HIA titers were defined as the reciprocal of the highest serum dilution that completely inhibited HA. Seroprotection was defined as an HIA titer ≥ 40. Seroconversion was defined as either an HIA titer < 10 before vaccination (D0) and ≥ 40 afterward or as an HIA titer ≥ 10 before vaccination followed by a ≥ 4-fold increase after vaccination.

### Phenotyping by spectral flow cytometry.

A 30-color panel was developed to evaluate frozen whole blood cell populations at D–7, D0 ,and D1 (Supplemental Table 12). Briefly, whole blood preserved in Cytodelics buffer was lysed and fixed following kit instructions, and it was sequentially incubated with anti-CX3CR1, Human TruStain FcX, and True-Stain Monocyte Blocker; other anti-chemokines (CCR5, CCR2, and CXCR4); and a mix of all other antibodies. The stained cells were fixed with 1% paraformaldehyde (Sigma-Aldrich) before being acquired on Aurora spectral cytometer (Cytek Biosciences) and analyzed using FlowJo v10.8.1 (BD Biosciences). During the monitoring, 1 patient receiving QIV-HD (patient 01-007-BB) displayed a very high percentage of B cells at D–7, D0, and D1. After review by the trial scientific committee, we decided to maintain the patient in the full analysis set. We analyzed the abundance of different blood cell subpopulation as indicated in the gating strategy (Supplemental Figure 2).

### Transcriptomic analysis.

For transcriptomic analysis, 2.5 mL whole blood was collected in PAXgene tubes (Qiagen) from each patient before vaccination (D–7and D0) and 24 hours later (D1). Total RNA was extracted from blood according to the handbook of the PAXgene Blood RNA Kit (PreAnalytiX). Purity and integrity of the RNA was assessed on the Agilent 2100 Bioanalyzer with the RNA 6000 Nano LabChip reagent set (Agilent). One V2 sample (no. 02-066-FB) of QIV-SD group without good RNA quantity was removed for transcriptomic analyze.

Sample preparation for microarray hybridization was carried out as described in the Applied Biosystems GeneChip Whole Transcript (WT) PLUS Reagent Kit User Guide (Thermo Fisher Scientific), and RNA was hybridized to Applied Biosystems GeneChip Clariom S human arrays. The fluorescence signals were measured with an Applied Biosystems GeneChip GeneChip Scanner 3000 7G System and were controlled by the Applied Biosystems GeneChip Command Console v5.0 software. Affymetrix’ Guanine Cytosine Count Normalization algorithm and Affymetrix’ Signal Space Transformation algorithm were used to normalized raw data by using Affymetrix Power Tools software v2.11.4.

Based on the quality control procedures, 13,427 probe sets (72.2%) were considered noninformative. They were excluded when performing the unsupervised analyses, leaving 5,174 remaining probe sets.

To decrease the influence of outlier samples on the differential expression analysis of transcriptomes, we estimated relative quality weights for each array and injected them into the linear regression model for microarray data evaluating the relative measures of mRNA expression level for each gene, by trial arm: Y(*Gene*)*_i_* = β0 + βVaccine*_j_* + ϵ. With Y(*Gene*)*_i_* as the gene *i* of interest (response variable), β_0_ the model intercept, βVaccine*_j_* the effect of allocated vaccine *j* (QIV-HD or QIV-SD) on gene expression, and ϵ model residuals.

Comparisons of interest were computed through statistical contrasts for the following: QIV-SD|(D1 – D0), QIV-HD|(D1 – D0). The empirical Bayes method was used to compute moderated *P* values. We then corrected *P* values for multiple comparisons using the Benjamini and Hochberg’s FDR controlling procedure. Genes declared noninformative by the informative/noninformative method were first filtered out. We displayed genes that are differentially expressed using s, considering adjusted *P* < 0.05, and FC > 1.41 or < 1/1.41, to indicate significance.

### Meta-analysis.

Three previous studies have studied the gene expression changes in the whole blood of young adults D1 after inactivated influenza vaccination. The available transcriptomics data were downloaded from the Gene Expression Omnibus (GEO) repository. The GSE30101 study included 6 adults aged 18–64 years. The GSE48023 included 128 healthy female volunteers aged 19–41 years, and the GSE48018 recruited 119 healthy male volunteers aged 19–41 years. In these 3 studies, volunteers received the seasonal Trivalent influenza vaccine.

For each study, data were downloaded from the GEO database, and samples from D0 (baseline) and D1 after vaccination were selected. Data were log-transformed and quantile normalized, and the R package *limma* was used to retrieve the genes differentially expressed between day 1 and baseline. Tables provided by *limma* for each study were used as input for the meta-analysis, performed with the *MetavolcanoR* package, developed to combine various differential expression analysis results. The combining approach from the *MetavolcanoR* package was used to summarize the FC of the studies based on the mean of the values, while the *P* value was combined using the Fisher method.

### Enrichment.

Using the *limma*’s output for groups QIV-SD and QIV-HD (elderly) groups and the ones from meta-analysis in young adults, genes were ranked based on their *P* values. Enrichment analyses were performed using the blood transcription modules (BTM) gene set, and the significance of each enriched module was assessed by the CERNO test, a multivariate ranked test provided by the *tmod* R package.

### Data visualization.

The *ggplot2* R package was used for visualization of differential expression analysis results of groups QIV-SD and QIV-HD, as well as the comparison of the FC in these groups. *Hclust*, *Pheatmap*, and *ComplexHeatmap* R packages were used for clusterization of data and visualization using the heatmap representation of the log_2_ FC values of different genes linked to IFN and antiviral responses. HIA titer and cell population analyses were performed with GraphPad Prism 10.

### Statistics.

Baseline characteristics of the study population were expressed as frequency (percentages) for qualitative variables and as medians (with interquartile range) for continuous variables. Geometric means and their 95% CI were computed for HIA titers.

The full analysis set (*n* = 59) used for statistical analysis encompassed the intention-to-treat population (i.e., all randomized participants). We compared baseline characteristics between the groups with χ^2^ or Fisher’s exact tests for qualitative variables, and with Kruskal-Wallis or Mann-Whitney *U* tests for quantitative variables. Evolution of HIA titers from D0 to D21 between groups used a Wilcoxon matched-pairs signed rank test.

For cell populations, statistical analyses were performed with 1-way ANOVA and Bonferroni’s multiple-comparison tests.

Spearman was the chosen method for correlation analysis, which used as input data in log_2_FC to transcriptomics, immune cells, and antibody titers. Correlation analyses using the 293 DEG were performed using all the samples in order to understand how the shared signatures found among the groups, and they were linked to the immune responses observed; a filter of *P* < 0.05 was used. In order to find specific signatures for the high-dose group, a correlation analysis using the whole gene expression dataset for the QIV-HD group and the A/H3N2 HIA titers was performed after filtering out the 25%-less variant genes; features with *P* < 0.05 and an absolute value of *r* > 0.4 were considered significantly correlated.

All statistical analyses were performed using the R software (R for statistical computing, The R foundation), with the *limma* and *stat* packages for transcriptomic analysis. A 5% bilateral threshold indicated significance.

### Study approval.

The Influomics trial was conducted in accordance with the Declaration of Helsinki and the International Conference on Harmonization Good Clinical Practice guidelines. It was approved by relevant Health authority (Agence National de Sécurité du Médicament et des produits de Santé, notification MEDAECNAT-2021-08-0021) and an independent ethics committee (Comité de Protection des Personnes, CPP Ile de France III Paris, #21.02400.000018). Trial registration included EudraCT no. 2021-004573-32. Each patient provided written informed consent before participation in the trial.

### Data availability.

All data analyzed in this study are included in the published article. Values for all graphs and supplemental graphs are reported in the Supporting Data Values file. The normalized microarray data that support the finding of this study have been deposited in ArrayExpress with the accession code E-MTAB-14109.

## Author contributions

All authors contributed to the article and approved the submitted version. OB and TD share first authorship in the given order based on their relative contributions to the project. BC and CJ share last authorship in the given order based on their relative contributions to the project. CJ, BC, and BL conceptualized of this project. CJ and BC provided resources and funding for the project. MG was responsible for project administration. TD and BC prepared the methodology of the study. CJ, OR, and BC were involved in investigation project. OB, JN, KA, SDB, BL, CJ, MG, MP, and TD were involved in data acquisition and validation. OB, IFM, SB, MW, BC, SDB, KA, JN, MP, TD, and BL performed formal analysis. OB, BC, TD, and CJ supervised and managed data and acquisition of resources. TD, MG, ML, MP, and BL were involved in data curation. OB, BC, IFM, and KA were involved in data visualization, and BC, OB, IFM, TD, and CJ in writing and original and revision draft preparation. The INFLUOMICS Study group was involved in investigation and in data acquisition. The group also performed formal analysis, managed data and acquisition of resources, and was involved in data curation.

## Supplementary Material

Supplemental data

ICMJE disclosure forms

Supplemental tables 5-11

Supporting data values

## Version 1 03/04/2025

In-Press Preview

## Version 2 04/22/2025

Electronic publication
