# Supplementary material for: Boosting effect of high-dose influenza vaccination on innate immunity among elderly
Source: JCI Insight. 2025 Mar 4;10(8):e184128. doi: 10.1172/jci.insight.184128 (PMC12016920; doi:10.1172/jci.insight.184128)
Supplement: Supplemental data [file jciinsight-10-184128-s268.pdf]

Supplemental Figure S1: Flow diagram of 60 participants of Influomics cohort. Flow of participants through the Influomics clinical trial, according to Consolidated Standard of Reporting Trials (CONSORT). Sixty participants were enrolled, randomized in 2 arms. QIV-HD: high-dose quadrivalent influenza vaccine; QIV-SD: standard-dose quadrivalent influenza vaccine. Ph.Ex: Physical examination.

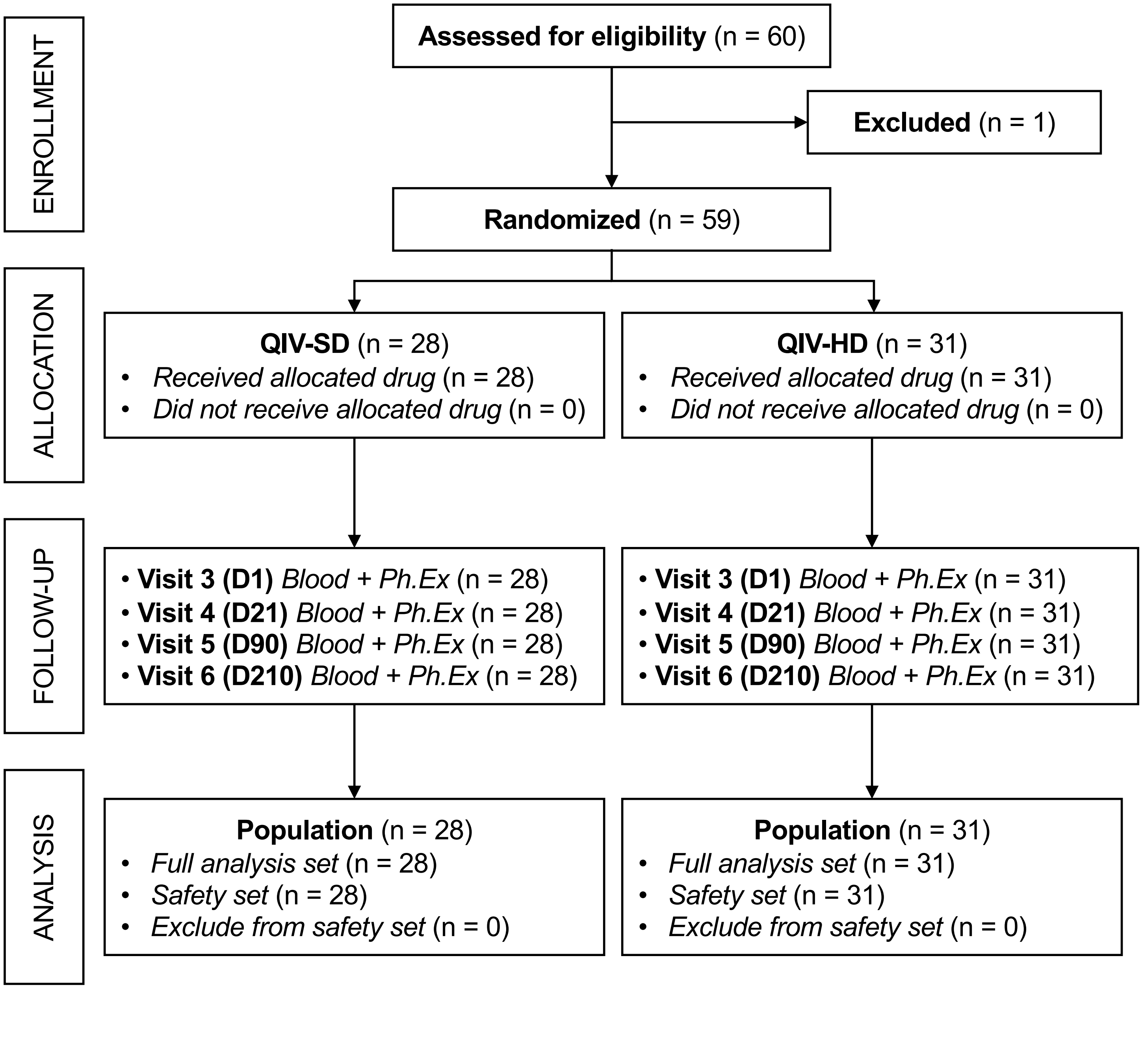

Supplemental Figure S2: Representative gating strategy for identification of whole blood cell populations. Natural killer (NK) cells; plasmacytoid (pDCs), conventional 1 (cDC1) conventional 2 (cDC2) and monocyte-derived (moDCs) dendritic cells.

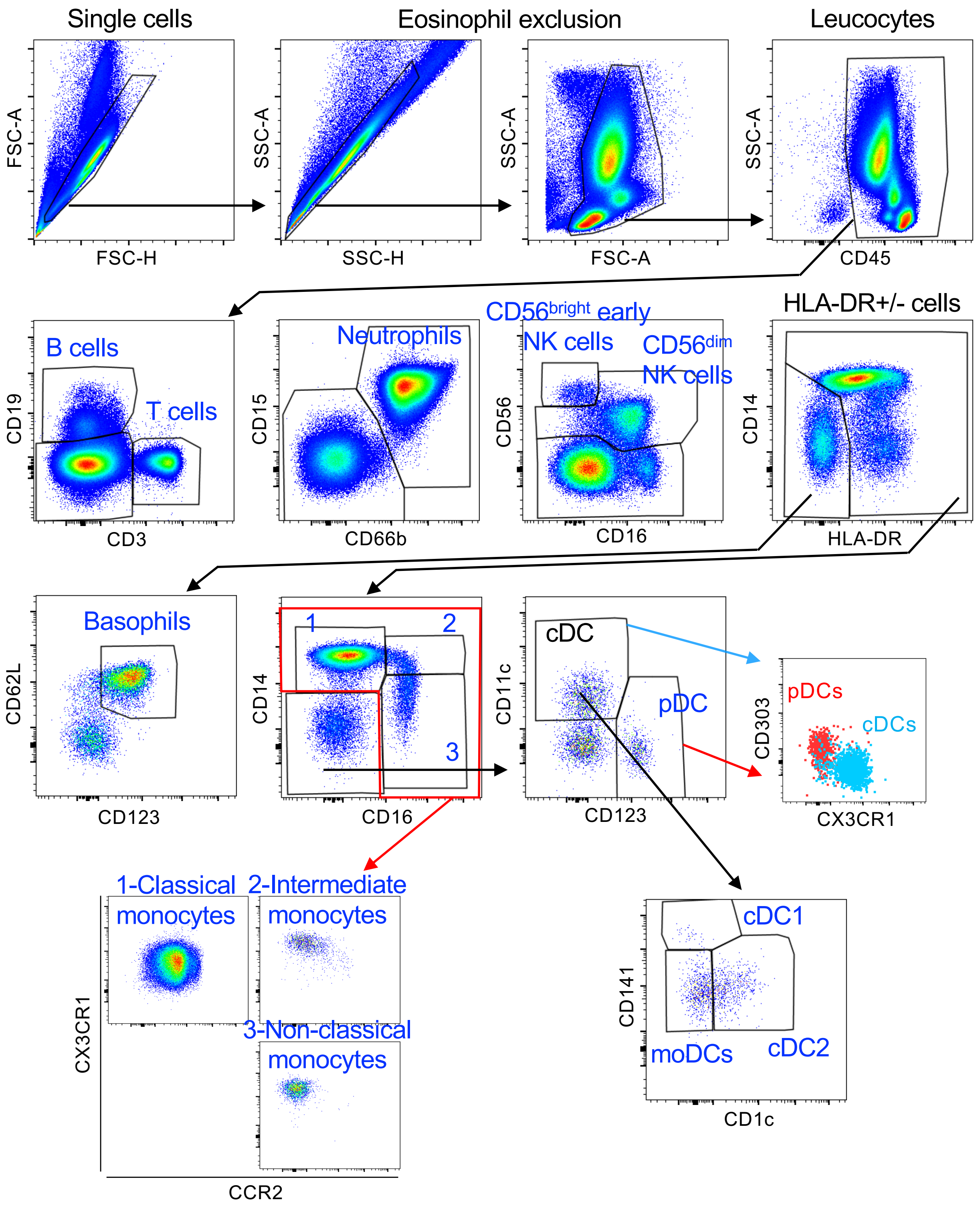

Supplemental Figure S3: Baseline stability of whole blood cell populations. Plots show percent of blood populations of each subject in QIV-SD (green) and QIV-HD group (violet) before vaccination (Day-7 (D-7) and D0): T cells, B cells, Neutrophils, Basophils; CD56^{bright} early and CD56^{dim} NK cells; classical, intermediate and non-classical Monocytes; and plasmacytoid (pDC), conventional 1 (cDC1), conventional 2 (cDC2) and monocyte-derived (moDC) Dendritic Cells. Wilcoxon matched-pairs signed rank test were performed to compare the two baseline of each group.

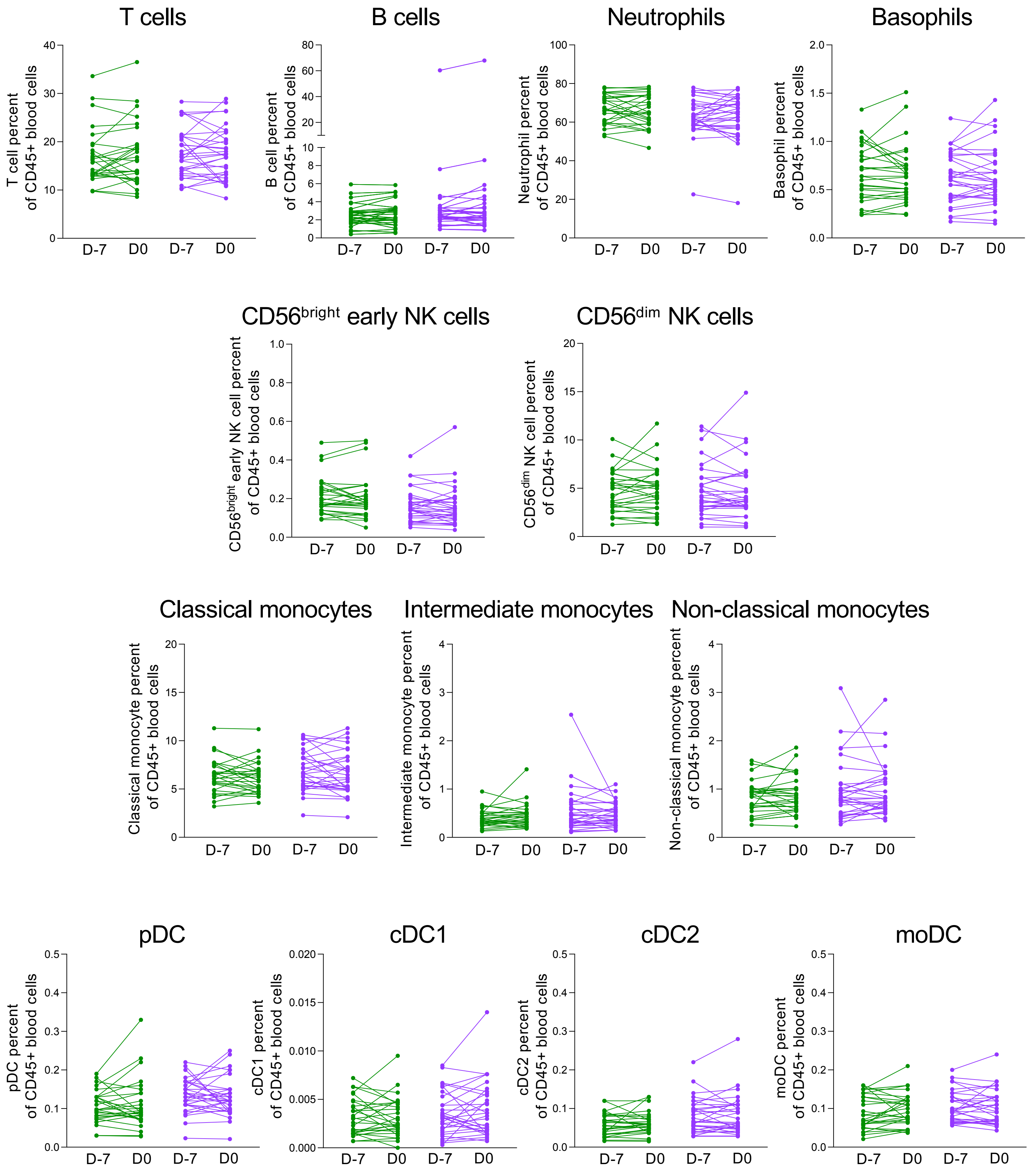

Supplemental Figure S4: Circulating blood cell populations after vaccination. Violin plots of fold change D1/D0 (Log2) are represented for T cells, B cells, neutrophils, basophils, CD56^{dim} natural killer (NK) cells, classical, intermediate and non-classical monocytes, plasmacytoid (pDCs), conventional dendritic cells type 1 (cDC1), and monocyte-derived DC (moDCs) (from top to bottom and right to left respectively) of QIV-SD (green) and QIV-HD (violin) subject groups. Statistical analyses were performed with one-way ANOVA and Bonferroni's multiple comparison tests.

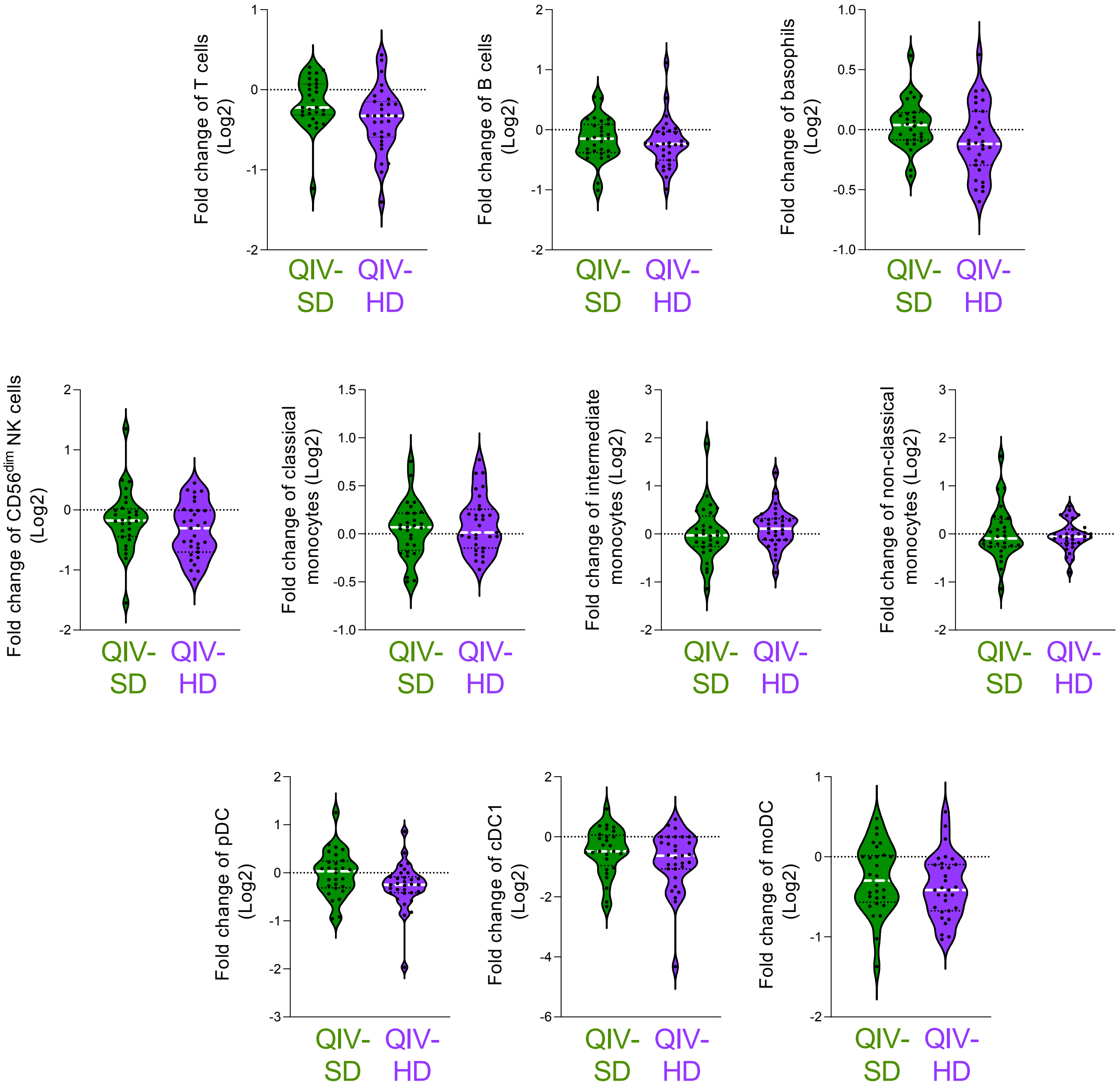

Supplemental Figure S5: Significant up- and down-regulated gene associated with NK cell response in elderly vaccinated with QIV-SD and QIV-HD compared to young adults vaccinated with TIV-SD.

Tmod enrichment analysis. From the list of genes provided by the DESeq2 package, genes were selected by their False discovery rate (FDR) p-value (< 0.05) and absolute log2 Fold-Change (> 0.49). The enriched blood transcription modules were obtained by the Hypergeometric test. Modules related to NK cell response. The effect size is proportional to the size of the pie, while the adjusted p-value is proportional to color intensity. Within each pie, the proportion of significantly upregulated and downregulated genes is shown in red and blue, respectively. The grey portion of the pie represents genes that are not significantly differentially regulated.

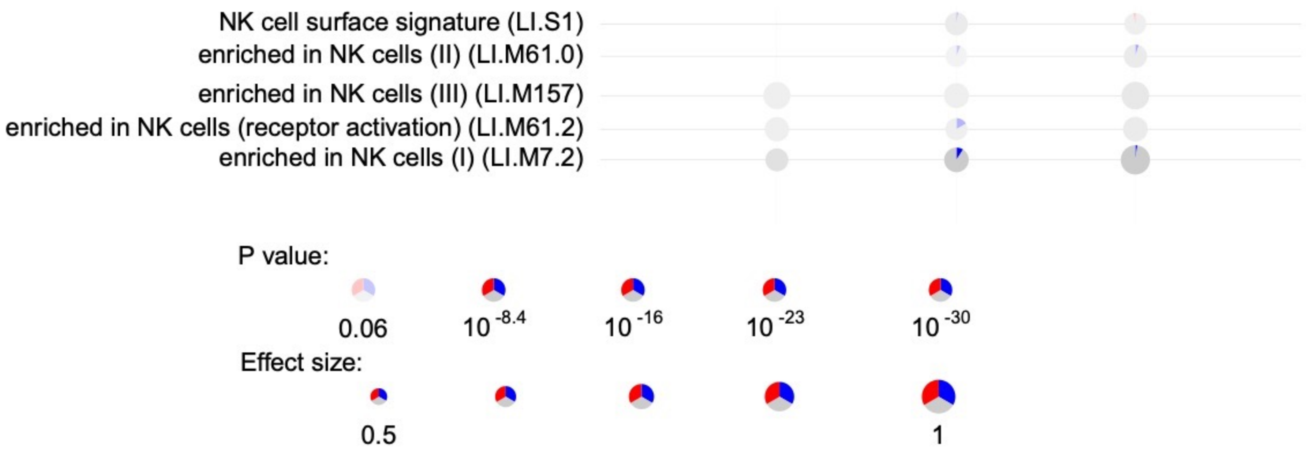

Supplemental Table S1: Demographic statue of 59 participants of Influomics cohort. QIV-HD: high-dose quadrivalent influenza vaccine; QIV-SD: standard-dose quadrivalent influenza vaccine; [1]Age-adjusted Charlson comorbidity score

Variable	QIV-SD (N = 28)		QIV-HD (N = 31)		Overall	
	N / median	*% / (IQR)*	*N / median*	*% / (IQR)*	*N / median*	*% / (IQR)*
Age (years)	70	(67 - 72)	70	(69 - 74)	70	(68 - 73)
Gender, *female*	11	39%	14	45%	25	42%
Body Mass Index (BMI)	25	(23 - 27)	24	(23 - 26)	24	(23 - 27)
Charlson Comorbidity Index[1] (CCI)	3.0	(2.0 - 4.0)	3.0	(3.0 - 4.0)	3.0	(2.0 - 4.0)
Myocardial infarction, *yes*	2	7.1%	0	0.0%	2	3.4%
Congestive heart failure, *yes*	0	0.0%	0	0.0%	0	0.0%
Peripheral vascular pathology, *yes*	5	18%	7	23%	12	20%
Chronic lung disease, *yes*	1	3.6%	5	16%	6	10%
Dementia, *yes*	0	0.0%	0	0.0%	0	0.0%
Oeso-gastro-dueodenal ulcers, *yes*	0	0.0%	0	0.0%	0	0.0%
Hepatopathies						
Mild	1	3.6%	0	0.0%	1	1.7%
Moderate to severe	0	0.0%	0	0.0%	0	0.0%
Chronic kidney disease - moderate to severe, *yes*	0	0%	1	3.2%	1	1.7%
Diabetes						
Uncomplicated	2	7.1%	0	0.0%	2	3.4%
Complicated	0	0.0%	0	0.0%	0	0.0%
Connective tissue diseases, *yes*	0	0.0%	0	0.0%	0	0.0%
Hemiplegia, *yes*	0	0.0%	0	0.0%	0	0.0%
Cancer						
No	25	89%	29	94%	54	92%
Yes, current treatment or less than 3 months old	1	3.6%	0	0.0%	1	1.7%
Yes, without treatment for more than 3 years	2	7.1%	2	6.5%	4	6.8%
Leukemia, *yes*	0	0.0%	0	0.0%	0	0.0%
Lymphoma, *yes, without treatment for more than 3 years*	0	0.0%	1	3.2%	1	1.7%
Solid tumor, *yes*	0	0.0%	0	0.0%	0	0.0%
AIDS, *yes*	0	0.0%	0	0.0%	0	0.0%
Other medical conditions at enrollment						
Heart rhythm disorders, *yes*	3	11%	4	13%	7	12%
Psychic assignments, *yes*	2	7.1%	4	13%	6	10%
Inflammatory digestive diseases, *yes*	0	0.0%	0	0.0%	0	0.0%
Auto-inflammatory/auto-immune diseases, yes	0	0.0%	0	0.0%	0	0.0%
Asplenia	0	0.0%	0	0.0%	0	0.0%
Hypercholesterolemia, dyslipidemia, *yes*	9	32%	9	29%	18	31%
Multiple myeloma	0	0.0%	0	0.0%	0	0.0%
Headaches, *yes*	2	7.1%	2	6.5%	4	6.8%
Dizziness, *yes*	1	3.6%	2	6.5%	3	5.1%
Dermatological conditions, *yes*	5	18%	5	16%	10	17%
Ongoing anti-inflammatory treatment, *yes*	0	0%	3	9.7%	3	5.1%
Smoking						
Active smoker	3	11%	0	0.0%	3	5.1%
Former smoker	11	39%	19	61%	30	51%
Non-smoker	14	50%	12	39%	26	44%

Supplemental Table S2: Historical flu and SARS-CoV-2 vaccinations of 59 participants of Influomics cohort. QIV-HD: high-dose quadrivalent influenza vaccine; QIV-SD: standard-dose quadrivalent influenza vaccine.

Variable	QIV-SD (N = 28)		QIV-HD (N = 31)		Overall	
	N / median	*% / (IQR)*	*N / median*	*% / [IQR]*	*N / median*	*% / [IQR]*
Vaccination against flu during previous season						
Vaccination, *yes*	23	82%	26	84%	49	83%
Time since last injection (months)	13.0	(13.0 - 13.0)	13.0	(12.0 - 13.0)	13.0	(12.0 - 13.0)
SARS-CoV-2 infection prior inclusion						
Infection, *yes*	2	7.1%	6	19%	8	14%
Time since infection (months)	11.0	(6.5 - 16.0)	13.0	(11.0 - 14.0)	13.0	(11.0 - 16.0)
Vaccination against SARS-CoV-2 prior inclusion						
Vaccination, *yes*	28	100%	31	100%	59	100%
Time since last injection (months)	2.0	(1.0 - 5.3)	3.0	(1.0 - 5.0)	2.0	(1.0 - 5.0)

Supplemental Table S3: Safety of 59 participants of Influomics cohort. QIV-HD: high-dose quadrivalent influenza vaccine; QIV-SD: standard-dose quadrivalent influenza vaccine; AE: adverse event, SAE: serious adverse event.
Five SAE were reported of which two occurred in the same patient. The occurrence of Ecchymotic lesions on injection site was considered related to the vaccine. The other SAEs were not related to the vaccine.

Type of AE	QIV-SD (N=28)	QIV-HD (N=31)
Number of patients with at least one AE	0 (0%)	0 (0%)
Number of patients with at least one SAE		
- None	27 (96.4%)	28 (90%)
- At least one SAE	1 (3.6%)	3 (10%)

Supplemental Table S4: Flu-specific antibody response of 59 participants of Influomics cohort. Geometric mean titers (GMT) of hemagglutination inhibition antibodies (HIA) and Geometric mean of individual titer ratios (GMTR) and 95% confidence intervals (CI) on day 0 (D0) and D21 are shown for each QIV-SD and QIV-HD groups (respectively n=28 and n=31). Seroprotection rates (i.e., numbers of individuals with HIA titers ≥40) and seroconversion rates (i.e., numbers of individuals with HIA titers <10 at D0 and HIA titers ≥40 after vaccination or with HIA titers ≥10 at D0 and ≥4-fold increase in HIA titers after vaccination) are also indicated. Wilcoxon tests and Fisher's exact tests were performed to compare the characteristics of the two groups (*p-value<0.05; **p-value<0.01; ***p-value<0.0).

		QIV-SD (N = 28)		QIV-HD (N = 31)		p value
Influenza A/H1N1						
Day 0	GMT (95% CI)	38.2	(25.5 - 50.9)	38.7	(24.8 - 52.6)	0.895
	Seroprotection, n (%)	13 (46.4%)	(19.3 - 73.5)	13 (41.9)	(15.1 - 68.8)	1.000
Day 21	GMT (95% CI)	302.1	(118.6 - 485.6)	546.5	(353.6 - 739.4)	<0.01**
	Seroprotection, n (%)	26 (92.9%)	(82.9 - 100.0)	31 (100.0%)	(100.0 - 100.0)	0.220
	Seroconversion, n (%)	14 (50.0%)	(23.8 - 76.2)	31 (100.0%)	(100.0 - 100.0)	<0.001***
	GMTR (95% CI)	16.6	(3.9 - 29.3)	31.5	(7.4 - 55.6)	<0.01**
Day 90	GMT (95% CI)	259.2	(178.1 - 340.3)	372.9	(267.9 - 477.9)	0.112
	Seroprotection, n (%)	25 (89.3%)	(77.2 - 100.0)	30 (96.8%)	(90.5 - 100.0)	0.337
	Seroconversion, n (%)	20 (71.4%)	(51.6 - 91.2)	30 (96.8%)	(90.4 - 103.1)	<0.01**
	GMTR (95% CI)	11.3	(6.4 - 16.2)	18.5	(6.3 - 30.7)	0.124
Day 210	GMT (95% CI)	199.1	(132.0 - 266.2)	222.9	(156.3 - 289.5)	0.521
	Seroprotection, n (%)	24 (85.7%)	(71.7 - 99.7)	29 (93.5%)	(84.6 - 100.0)	0.409
	Seroconversion, n (%)	16 (57.1%)	(32.9 - 81.2)	21 (67.7%)	(47.7 - 87.7)	0.431
	GMTR (95% CI)	7.6	(4.8 - 10.4)	10.8	(2.8 - 18.8)	0.506
Influenza A/H3N2						
Day 0	GMT (95% CI)	70.7	(49.5 - 91.9)	131.7	(43.6 - 219.8)	0.908
	Seroprotection, n (%)	19 (67.9%)	(46.9 - 88.9)	19 (61.3%)	(39.4 - 83.2)	1.000
Day 21	GMT (95% CI)	306.5	(95.0 - 518.0)	667.7	(280.5 - 1055)	<0.01**
	Seroprotection, n (%)	22 (78.6%)	(61.4 - 95.7)	31 (100.0%)	(100.0 - 100.0)	<0.01**
	Seroconversion, n (%)	10 (35.7%)	(6.0 - 65.4)	20 (64.5%)	(43.5 - 85.4)	0.037*
	GMTR (95% CI)	10.6	(- 2.8 - 24.0)	48.7	(- 23.7 - 121.1)	<0.001***
Day 90	GMT (95% CI)	256.4	(156.3 - 356.5)	643.2	(325.1 - 961.3)	0.077
	Seroprotection, n (%)	25 (89.3%)	(77.2 - 100.0)	31 (100.0%)	(100.0 - 100.0)	0.100
	Seroconversion, n (%)	12 (42.9%)	(14.9 - 70.9)	24 (77.4%)	(60.7 - 94.1)	<0.01**
	GMTR (95% CI)	8.6	(- 0.2 - 17.4)	32.0	(- 16.1 - 80.1)	<0.01**
Day 210	GMT (95% CI)	198.4	(128.1 - 268.7)	434.8	(221.8 - 647.8)	0.192
	Seroprotection, n (%)	23 (82.1%)	(66.5 - 97.8)	29 (93.5%)	(84.6 - 100.0)	0.239
	Seroconversion, n (%)	10 (35.7%)	(6.0 - 65.4)	17 (54.8%)	(31.1 - 78.5)	0.192
	GMTR (95% CI)	5.5	(1.1 - 9.9)	21.4	(- 10.7 - 53.5)	0.035*
Influenza B/Victoria						
Day 0	GMT (95% CI)	13.4	(8.4 - 18.4)	10.8	(5.6 - 16.0)	0.616
	Seroprotection	5 (17.9%)	(0.0 - 51.4)	2 (6.5%)	(0.0 - 40.5)	0.239
Day 21	GMT (95% CI)	53.0	(24.5 - 81.5)	63.4	(36.8 - 90.0)	0.107
	Seroprotection, n (%)	14 (50.0%)	(23.8 - 76.2)	18 (58.1%)	(35.3 - 80.9)	0.605
	Seroconversion, n (%)	7 (25.0%)	(- 7.1 - 57.1)	19 (61.3%)	(39.4 - 83.2)	<0.01**
	GMTR (95% CI)	5.8	(1.7 - 9.9)	7.7	(4.8 - 10.6)	<0.01**
Day 90	GMT (95% CI)	54.1	(29.4 - 78.8)	74.5	(41.0 - 108.0)	0.263
	Seroprotection, n (%)	14 (50.0%)	(23.8 - 76.2)	21 (67.7%)	(47.7 - 87.7)	0.193
	Seroconversion, n (%)	13 (46.4%)	(19.3 - 73.5)	23 (74.2%)	(56.3 - 92.1)	0.035*
	GMTR (95% CI)	5.1	(2.7 - 7.5)	9.1	(5.1 - 13.1)	0.019*
Day 210	GMT (95% CI)	43.8	(21.1 - 66.5)	49.0	(27.5 - 70.5)	0.449
	Seroprotection, n (%)	13 (46.4%)	(19.3 - 73.5)	18 (58.1%)	(35.3 - 80.9)	0.438
	Seroconversion, n (%)	11 (39.3%)	(10.4 - 68.1)	20 (64.5%)	(43.5 - 85.4)	0.069
	GMTR (95% CI)	3.8	(2.3 - 5.3)	5.9	(3.6 - 8.2)	0.066
Influenza B/Yamagata						
Day 0	GMT (95% CI)	12.5	(8.4 - 16.6)	14.8	(8.6 - 21.0)	0.882
	Seroprotection, n (%)	3 (10.7%)	(0.0 - 45.7)	6 (19.4%)	(0.0 - 50.9)	0.477
Day 21	GMT (95% CI)	41.1	(21.6 - 60.6)	48.3	(30.7 - 65.9)	0.307
	Seroprotection, n (%)	11 (39.3%)	(10.4 - 68.1)	13 (41.9%)	(15.1 - 68.8)	1.000
	Seroconversion, n (%)	5 (17.9%)	(- 15.7 - 51.4)	12 (38.7%)	(11.1 - 66.2)	0.092
	GMTR (95% CI)	4.5	(1.0 - 8.0)	4.8	(2.6 - 7.0)	0.196
Day 90	GMT (95% CI)	46.4	(27.1 - 65.7)	42.8	(26.9 - 58.7)	0.800
	Seroprotection, n (%)	12 (42.9%)	(14.9 - 70.9)	13 (41.9%)	(15.1 - 68.8)	1.000
	Seroconversion, n (%)	8 (28.6%)	(- 2.7 - 59.9)	12 (38.7%)	(11.1 - 66.3)	0.582
	GMTR (95% CI)	4.5	(1.9 - 7.1)	3.5	(2.4 - 4.6)	0.449
Day 210	GMT (95% CI)	36.2	(22.2 - 50.2)	32.4	(20.4 - 44.4)	0.799
	Seroprotection, n (%)	11 (39.3%)	(10.4 - 68.1)	11 (35.5%)	(7.2 - 63.8)	0.793
	Seroconversion, n (%)	7 (25.0%)	(- 7.1 - 57.1)	6 (19.4%)	(- 12.3 - 51.0)	0.755
	GMTR (95% CI)	3.3	(1.9 - 4.7)	2.5	(1.8 - 3.2)	0.730

Supplemental Table S12: Reagents for spectral cytometric analyses

Reagent	Clone	Source	Catalogue number
Mouse anti-human CD16 BUV496	3G8	BD Biosciences	612944
Mouse anti-human CD195 (CCR5) BUV563	2D7	BD Biosciences	741401
Rat anti-human CX3CR1 BUV615	2A9-1	BD Biosciences	751513
Mouse anti-human CD1c BUV661	F10/21A3	BD Biosciences	750181
Mouse anti-human CD56 BUV737	NCAM16.2	BD Biosciences	612766
Mouse anti-human CD11c BUV805	B-ly6	BD Biosciences	742005
Mouse anti-human CD26 BV421	M-A261	BD Biosciences	744448
Mouse anti-human CD123 SuperBright436	6H6	eBiosciences	62-1239-42
Mouse anti-human CD66b V450	G10F5	BD Biosciences	561649
Mouse anti-human BDCA-2 (CD303) BV480	V24-785	BD Biosciences	748006
Mouse anti-human CD11b BV510	ICRF44	BD Biosciences	563088
Mouse anti-human CD3 BV570	UCHT1	BioLegend	300436
Mouse anti-human CD169 BV605	7-239	BioLegend	346010
Mouse anti-human CD62L BV650	DREG-56	BioLegend	304831
Mouse anti-human CD163 BV711	GHI/61	BioLegend	333629
Mouse anti-human CD15 BV750	W6D3	BD Biosciences	747426
Mouse anti-human CD36 BV785	CLB-IVC7	BD Biosciences	745554
Mouse anti-human CD141 BB515	1A4	BD Biosciences	565084
Mouse anti-human CD14 SparlBlue550	63D3	BioLegend	367148
Mouse anti-human CD45 PerCP	2D1	BioLegend	368506
Mouse anti-human CD192 (CCR2) PerCP-Cy5.5	K036C2	BioLegend	357204
Mouse anti-human LOX-1 PE	15C4	BioLegend	358604
Mouse anti-human CD274 (PD-L1) PE/Dazzle594	29E.2A3	BioLegend	329731
Mouse anti-human CD10 PE/Cy5	HI10a	BioLegend	312206
Mouse anti-human CD184 (CXCR4) PE/Cy7	12G5	BioLegend	306513
Mouse anti-human MERTK APC	590H11G1E3	BioLegend	367612
Mouse anti-human CD19 SparkNIR685	HIB19	BioLegend	302269
Mouse anti-human CD64 AF700	10.1	BD Biosciences	561188
Mouse anti-human CD69 APC-H7	FN50	BD Biosciences	560737
Mouse anti-human HLA-DR APC/Fire810	L243	BioLegend	307674
True-Stain Monocyte Blocker		BioLegend	426103
Human TruStain FcX		BioLegend	422302
Whole blood processing kit /Gen 2		Cytodelics	hC002-1000
Paraformaldehyde solution 36%		Sigma-Aldrich	47608

Supplemental Acknowledgments

INFLUOMICS study group members not listed in the author line includes :

Antonin Bal[1,2], Lisa Barrandon[5], Silvère Biavat[4], Maude Bouscambert-Duchamp[1,2], Hélène Bricout[6], Pauline Campestrin[5], Marie Christine Carret[3], Ophélie Dos Santos[4], Marion Fournier[6], Jacques Gaillat[5], Alexandre Gaymard[1,2], Blandine Lafitte[5], Marie Cécile Levant[6], Gabriel Macheda[5], Sophie Mugnier[5], Margaux Ortonne[5], Lucas Pires[5], Sandrine Samson[6], Philippe Travers[4], Martine Valette[1,2]

1 Centre International de Recherche en Infectiologie, Université Claude Bernard Lyon-1, INSERM U1111, CNRS, UMR5308, ENS Lyon, Université Jean Monnet de Saint-Etienne - Lyon, France.

2 Laboratoire de Virologie, Institut des Agents Infectieux, Centre National de Référence des Virus des Infections Respiratoires, Hospices Civils de Lyon, F-69317 Lyon Cedex 04, France.

3 Unité de recherche clinique, Centre Hospitalier Centre Hospitalier Métropole Savoie 73000 - Chambéry, France

4 Espace de Santé Publique, Centre Hospitalier Centre Hospitalier Métropole Savoie 73000 - Chambéry, France

5 Centre Hospitalier Annecy Genevois, 74330 - Epagny Metz-Tessy, France.

6 Sanofi Vaccins, Affaires Médicales, France
